# Creating and Sustaining Service Industry Relationships and Families: Theorizing How Personal Workplace Relationships Both Build Community and Perpetuate Organizational Violence

**DOI:** 10.3390/bs12060184

**Published:** 2022-06-08

**Authors:** Elizabeth K. Eger, Emily Pollard, Hannah E. Jones, Riki Van Meter

**Affiliations:** 1 Department of Communication Studies, Texas State University, San Marcos, TX 78666, USA; rikileigh14@gmail.com (R.V.M.); 2 Department of Communication and Journalism, University of New Mexico, Albuquerque, NM 87131, USA; epollard07@unm.edu; 3 Department of Communication, Rutgers, The State University of New Jersey, New Brunswick, NJ 08901, USA; hannah.jones@rutgers.edu

**Keywords:** personal workplace relationships, service industry, occupational identification, organizations as families, organizational violence, organizational communication, work-life, restaurant workers

## Abstract

Service industry workers experience challenging labor conditions in the United States, including pay below the minimum wage, expected emotional labor, and harassment. Additionally, in part because they work long shifts in high stress environments in restaurants and bars, many build and form personal workplace relationships (PWRs). In 2021, we interviewed 38 service industry workers and managers during the COVID-19 pandemic where we examined occupational challenges they faced in the state of Texas, USA. Through our interpretive research, this essay showcases our inductive findings on how service industry workers and managers utilize communication to create and sustain PWRs. We identified how some PWRs are sustained through a unique form of occupational identification that cultivates a “service industry family”, which we term familial personal workplace relationships (familial PWRs). This extends past organizational communication scholarship on family to consider occupational identification. Furthermore, our research reveals that while PWRs may build communities through care and support, they also perpetuate organizational violence, like sexual harassment and bullying.

## 1. Introduction

Through organizations and occupations, workers cultivate identities, relationships, and means of economic survival. Interdisciplinary research explores how workers navigate the interconnections of life and work across their relationships. While work-life and communication researchers have extensively theorized white-collar work(ers), occupations, and organizations (e.g., [1,2,3]), less scholarship considers blue-collar and service labor, thereby limiting our broader conceptualizations of work, organizations, and occupations in the United States and globally. Our essay examines an occupation with unique work-life and communication challenges: the service industry.

The service industry, which includes work in restaurants, bars, and hospitality, is consistently one of the largest occupations in the United States. According to the U.S. Bureau of Labor [4], there were approximately 11.2 million service industry workers nationwide employed in 2021 in food preparation and serving related occupations. Service industry workers are also consistently paid the minimum wage or below [5] and have a mean annual wage of $ 29,450 USD [4]. Furthermore, while some industries in the United States are beginning to recover from the ongoing, devastating impact of the COVID-19 pandemic, the food and alcohol-based service industry has continued to endure intense struggles [6,7]. Viral social media images portray service industry workers being mistreated by customers and/or leaving their jobs due to over work during the pandemic [7]. Past research also illustrates that service industry workers face incivility by customers, coworkers, and supervisors, and how their level of integration into their organization impacts their interpersonal interactions and stressors [8]. Due to the occupation’s size, impact, and growing organizational communication challenges, we need further research on the service industry. We designed interpretive research to investigate how participants experienced and communicated their occupational challenges in the service industry, including during the global pandemic, through semi-structured interviews.

Through qualitative inquiry and analysis, we surfaced unique, inductive findings not included in our interview guide where participants repeatedly communicated the importance of personal workplace relationships (PWRs). PWRs are characterized by relationships within the workplace that are informal, mutual, and consensual in nature. Individuals in PWRs possess a stronger emotional bond than other types of workplace relationships due to their personal connections, and such relationships are formed and sustained when individuals grow and interact as whole people with unique life experiences (see [9]). Through interpersonal communication, organizational communication, and interdisciplinary research, we argue that service industry workers are uniquely placed to form PWRs. We also respond to calls for research on PWRs outside of white-collar and professional workplaces, as recent literature points to how such scholarship has rarely explored blue-collar, manual, or service labor ([9], see [10] as an exception). Current literature about the service industry largely focuses on interactions with customers [11,12], and we seek to research PWRs with managers and coworkers.

Our essay showcases how service industry workers and managers utilize communication to create and sustain PWRs, including through a unique form of occupational identification that cultivates a “service industry family”, which we term “familial personal workplace relationships” or “familial PWRs”. We theorize our findings in relationship to past organizational communication scholarship on family as organizational identification [13,14,15,16] and extend this to consider occupational identification. Moreover, our research reveals that while PWRs may build community through care and support, they also perpetuate what interpersonal communication scholars have called “dark side” and/or “inappropriate” communication interactions sustained through organizational violence, like sexual harassment. In addition to vibrant, caring PWRs, our analysis illustrates pervasive occupational norms within the service industry that may masquerade as “normal” through PWRs. We write to surface such norms and provide new potential futures for service industry workers, including our participants.

Our essay proceeds as follows. First, we review relevant literature to frame our inductive analysis on PWRs, organizations and occupational identification and as families, and the dark side of PWRs as organizational violence. Second, we present our qualitative inquiry, design, and data analysis on the service industry in Texas, USA. Third, we narrate themes that arose during data analysis of PWRs in the service industry on familial PWRs. Finally, we end with our theoretical contributions, limitations, and suggestions for future research.

## 2. Literature Review

Our literature review connects often disparate areas of scholarship to ground our inductive analysis: (1) personal workplace relationships, (2) organizations as families and occupational identification, and (3) the dark side of PWRs as organizational violence.

### 2.1. Personal Workplace Relationships

Personal workplace relationships consist of relationships that transcend traditional organizational boundaries [17], can range in closeness from platonic to romantic [9], and are viewed as informal [18]. PWRs are characterized as voluntary and mutually reciprocated [19,20] among individuals interacting with each other as whole people rather than just as organizational members [17].

In a recent, extensive review where Horan et al. [9] coined PWRs, they examined past research on: how friendships are developed, maintained, and deteriorate [20,21]; benefits and difficulties associated with PWRs [22,23]; the effects of workplace friendships on interpersonal outcomes such as social support and organizational and interpersonal satisfaction [20]; and intimate platonic workplace friendships and “work spouses” [24,25]. When individuals engage in PWRs, they traditionally spend time with each other away from the workplace [21], allowing them to deepen their closeness. Thus, individuals in PWRs blend their work and life domains by combining personal and organizational role components to form relationships [26]. If such relationships deteriorate, individuals can face emotional stress, especially “isolation, frustration, and unhappiness” [21] (p. 337).

In organizations, PWRs can help boost employee morale [20,26] and reduce turnover [27]. Furthermore, when organizational members co-create organizational cultures that are “respectful of diverse identities and lifestyles” [28] (p. 265), workplace friendships can be cultivated across identities and diverse experiences. For example, Rumens examined gay, heterosexual, and bisexual male workplace friendships, including across aging processes [28] and as both sustaining and disrupting heteronormativity at work [29].

In the service industry where employees frequently report low organizational morale [7] and turnover rates are traditionally higher than other job types [4,8], having PWRs may help lessen these effects and make working in the service industry more enjoyable. Similar to other fast-paced and time-intensive occupations, restaurant workers are often expected to be constantly flexible and available for work [30], thereby their time for building personal relationships outside of work may be scarce.

Simultaneously, PWRs may be viewed by customers and managers as interfering with job productivity [26] or as workplace distractions [31]. For example, when individuals engage in workplace friendships, others may perceive the relationships to be unfair [32] and potentially disruptive to organizational procedures [23]. Yet, in the service industry where workers can potentially be promoted quickly, status differences may be less pronounced than in other occupations. Thus, research on blue-collar workers, working-class people, and service work is needed to theorize PWRs and expand whose work (and communication about their work) matters and is valued in scholarship (see, [30,33]).

### 2.2. Organizations as Family and Organizational and Occupational Identification

Our essay also uniquely connects literature not currently theorized in relationship to PWRs: organizations as families. We linked research on PWRs to theorizing organizational members and their organizations as self-described “family” via our analysis. Smith and Eisenberg’s [16] essay illuminated the tensions and relationships of power when communicating family as a metaphor for organizations in their Disney research. Organizations as family, then, are not only metaphorical but also rhetorical and constructed via policies and programs [15]. Kirby [15] (p. 477) called for future research of “how organizations talk about *themselves* when appropriating family-like roles… How do organizations justify their appropriation of family-like roles and blurring the public and the private”? Our essay responds to this call to understand what happens when organizational members mark their PWRs as familial, and how this connects to their organizational and occupational identities and identification.

Past research illustrates how organizational families are created through communication. Golden-Biddle and Rao [14] (p. 599) researched a large nonprofit (NPO), theorizing how organizational members that perceive one another as a “family of friends” reinforced members’ “desire to view themselves as members of this family”. For their participants, this led to tensions like conflict avoidance and navigating sustaining the “family of friends” identity but also needing to control volunteers as a “vigilant monitor”. Their work clarifies the complexities of sustaining organizational families. In previous research, Eger [13] connected organizational identities (how organizational members construct a collective identity) and organizational identification (members identifying with the values and goals of the organization) to members’ co-construction of their organization as family. Her research examined how a nonprofit organization serving transgender and nonbinary people cultivated a “chosen family” as an organizational identity. Importantly, organizational family identities are, “co-constructed and sustained by those served in NPOs” [13] (p. 276), meaning that community members served by nonprofits also shape the organizational identity through communication. Eger’s [13] (p. 277) essay calls for further research on the “consequences of ‘family’ in theory and practice”, to which our research responds in the context of a specific occupation (the service industry), rather than a specific organizational form (NPOs).

Thus, scholarship should examine how “the family” can be tied not only to organizations but also to occupations and occupational identification (e.g., belonging and identifying with an occupational group), which we seek to theorize. To do so, we build on scholarship of occupational identification as a central form of identification. Meisenbach [34] (p. 259) explains workers “are turning toward occupations and individual careers as sites of stability for their social identities”. For example, Steigenberger and Mirc’s [35] longitudinal study examined how for hospital employees after a hospital acquisition, their level of occupational identification shaped their experiences of organizational integration. Respondents who identified with their occupation faced larger challenges integrating in the acquisition, and some were more likely to consider exiting the organization.

Similarly, workers may view their occupational identification as their primary identification, over their organization. Ishii et al. [36]’s study of librarians discovered participants understood who they are as “librarians” as their primary identification, which changed in its specificity of sub-areas of their occupation depending on their communication partners. Ishii et al. [36] also examined how librarians challenged occupational stereotypes. Their research may be extended to consider how workers may combat occupational stereotypes through their occupational identification. Furthermore, Kingsford et al.’s [37] (p. 12) interviews with employment seeking and U.S. governmental assistant programs, occupational identification was one of the most common “identity anchors’’ for people navigating “a limited capacity to work, were looking for work, or talked about desires to work”. Thus, identifying with an occupation can be a central facet of people’s identities even when unemployed or unable to work, and we seek to understand how occupations may shape PWRs.

Our essay also extends new research by Hanners and Malvini Redden [30] (p. 5) who investigated how U.S. restaurants workers create occupational identities and address larger discourses of service work as framed as “dirty work” and undesirable labor. Like Hanners and Malvini Redden [30] (p. 12), we also orient our inquiry of the service industry at an occupational level; their metaphor analysis found that some participants used the metaphors of “family” to describe teamwork, dysfunctional communication with coworkers, and organizational norms for compliance. Hanners and Malvini Redden [30] (p. 13) noted how family metaphors in restaurant work became “personally meaningful, transcending work functions to create kinship among the staff” that can also “point to problematic organizational culture issues”. While their essay focused on restaurant workers’ communication with customers, we examine the PWRs cultivated among employees and managers as a unique form of familial occupational identification.

### 2.3. PWRs as Sustaning Organizational Violence

Finally, while much research highlights the benefits of PWRs (see [9]), PWRs can cultivate “dark side” and “inappropriate” relationships and even sustain organizational violence. Negative outcomes, such as bullying and harassment, can be theorized as dark side or inappropriate behaviors that occur in an otherwise appropriate relationship. Dark side communication and behaviors are considered dysfunctional, distressing, and disruptive and have both direct and indirect implications for individual exploitation [38]. While the dark side metaphor is popular in research, Long [39] (p. 318) critiqued this term for the “negative connotations associated with the word ‘dark’ historically have had and continue to have significant and vicious implications for people of color in America and worldwide... The binary of ‘light’ equals purity and ‘dark’ equates to deviance has been used to create and manipulate human divisions for generations”. To address his critique, previous research studying PWRs (see [40]) has framed this through Duck and VanderVoot’s [41] (in)appropriateness of behaviors frame. While the relationship itself may be appropriate within the organization, the behaviors associated with the relationship become inappropriate and have direct and indirect implications for individual exploitation. We thus connect dark side and inappropriate frames to theorize how PWRs can directly sustain organizational violence, such as workplace harassment and bullying (e.g., [42,43]).

Our conceptualization of organizational violence follows Harris’ theorizing [44,45] that violence becomes a structural feature of work sustained through our everyday communication. We understand organizational violence to be both structural and interpersonal, i.e., not only developed in individual relationships but also sustained through organizational structures. Organizational violence can occur through physical, sexual, and emotional assault; verbal and nonverbal communication that targets others; in structural policies and practices that erase, harm, or fail to protect workers; and in a myriad of other ways. Therefore, organizational violence is simultaneously enacted by organizations (such as exploitative working conditions or discriminatory policies) and/or by those *in* organizations (i.e., managers leveraging their power as supervisors to abuse their workers) (see [44]).

Organizations can specifically enact and support violence through policies, shifting away from solely holding individuals responsible to instead understand “the organization’s role in assault—accomplished through invisible reference to whiteness and heteronormativity” [44] (p. 589). Here, Harris reminds us that organizational violence is experienced in relationship to difference and power, such as navigating co-occurring violence of racism, sexism, homophobia, transphobia, and colonization. Organizational violence thus sustains the marginalization of working-class people, workers of color, LGBTQ+ workers, immigrant workers, women, and other communities. This even occurs throughout academia [45,46,47,48]. As Leslie [48] (p. 148) explains, “encounters with systemic violence” of whiteness can make people want to “flee” not only their bodies but also their occupations and professions, especially as these are, as Cruz [46] (p. 128) argues, “Not just a single encounter, But another encounter, amidst a multitude”. Workers are often called to perform emotional labor during experiences of organizational violence, especially as people of color, which creates a “pain of performative professionalism” [47] (p. 140) that falls “unevenly on different bodies” [45] (p. 149).

Moreover, organizational violence becomes normative in specific occupations. For example, Alfuqaha et al. [49] note the prevalence of “workplace violence” that healthcare providers experience, specifically during the ongoing COVID-19 pandemic. Doctors and nurses in Jordan reported that verbal abuse was the most prevalent form of workplace violence, and they recommended personal, organizational, and occupational structural support for providers facing workplace violence [49]. In their analysis of sexual harassment communication among manufacturing workers, Icenogle et al. [50] found that women in white-collar occupations more easily identified sexual harassment communication than women in blue-collar occupations. Icenogle et al.’s [50] discovery further illustrates the need for additional research on sexual harassment within non-white-collar occupations, such as our own. Bi et al. [51] (p. 11) specifically examined organizational violence of service industry customers in South Korea, and their survey results revealed that restaurant employees faced verbal and physical abuse and sexual harassment by customers. Such repeated abuse by customers led to burnout and health impacts [51] (p. 12).

We also notice how emotional labor becomes an expected performance in service work and can even become violent. Hanners and Malvini Redden [30] (p. 4) described how restaurant work emotional labor centers on how “so much of the job is focused on social performances designed to elicit positive emotions from patrons who may be emotionally demanding, impatient, or irritated from hunger”. Emotional labor also can perpetuate and/or become a form of organizational violence. Emotional labor is a “source of organizational suffering [in] expressing inauthentic emotion as a requirement of work” [52] (p. 350), and “organizational structures produce and enable emotional abuse at work” [52] (p. 353). Tracy et al. [43] also linked the presence of workplace bullying and emotional pain. Additionally, Kim and Leach [53] (p. 510) recently theorized the interconnections of emotional labor in leading to burnout in customer support work in a South Korean call center. Their supported hypotheses showcased that when customers used unjust communication such as “yelling” or “using condescending language”, this was “directly linked to an increase in emotional labor”. Kim and Leach’s [53] (p. 511) article noted how cultivating justice among managers and employees could assuage the burnout customer service workers face as tied to their expected emotional labor in response to unjust communication. Our research thus uniquely interconnects areas of organizational violence and emotional labor in PWRs in customer service work among employees and managers, rather than focusing on customers’ inappropriate communication and perpetuating organizational violence, as showcased by current scholarship [30,51,53].

Finally, just as workplace relationships can be vehicles toward organizational violence, including supporting bullying and (sexual) harassment, they may also help employees to resist organizational violence and make sense of harassment, bullying, and other forms of violence. Namin et al. [8] (p. 2) analyzed how individual employees used social support and relationships with managers to challenge workplace incivility (such as “verbal attacks, withholding important information, spreading rumors”) occurring in the service industry in Norway. D’Cruz and Noronha [54] researched the roles that workplace friendships with coworkers play in creating bystander interventions in workplace bullying and also connect it to Human Resources (HR) strategies. Their analysis of call center employees in India addressed how bystanders and their close workplace friends who were targets of bullying navigated joint responses to workplace bullying. 

We thus seek to further understand how PWRs may enact and sustain organizational violence in occupations like the service industry, including across employees’ identities. We now turn to our methods and how our qualitative inquiry enriches this literature.

## 3. Methods

The following section introduces our qualitative research design, our service industry participants, and our data analysis, including why we selected Texas, USA, for our research.

### 3.1. Research Design

This essay presents one facet of our overall service industry research during COVID-19. Our qualitative inquiry utilized semi-structured interviews with service industry workers and managers in Texas, USA, to examine multiple research questions, including our focus herein on: How do service industry workers communicate about their occupational norms and challenges, including bullying, harassment, emotional labor, and health impacts on their work-life?

In 2021, of the over 11.2 million workers nationwide in food preparation and serving related occupations, over 1 million of them worked in the state of Texas [4]. The service industry in Texas makes up 10% of employment in the state [6]. The State of Texas does not track service industry demographics; yet the U.S. Bureau of Labor [55] reported that the average age of service industry workers nationwide is 29.9. A total of 51% identify as women, 74.9% identify as white, 13.4% identify as Black and/or African American, 6.8% identify as Asian, and 27.5% identify as Hispanic and/or Latino [56]. We focused our recruitment to meaningfully include participants from all ages, races, ethnicities, disabilities, and sexualities, which we examine below.

We selected the Texas service industry because of the unique challenges during the COVID-19 pandemic where U.S. state and local laws varied extensively on mask ordinances, rules about bars and restaurants occupancy and openings, and state best practices. Service industry work during the COVID-19 pandemic was a central part of another of our research questions; here we only include pandemic experiences that are salient to our analysis of PWRs. Additionally, our research team was personally connected to the Texas service industry, as Emily worked in it for six years and Riki for seven years. Hannah also worked in the service industry for five years, and Elizabeth worked in retail customer service for one year and has chosen family with decades of service industry experiences, connecting our team’s own work-life to the tensions this essay examines.

After receiving IRB approval, we recruited participants from the Texas service industry in Fall 2020 and Spring 2021 using social media posts, social media food and beverage groups, our personal and professional networks, and hand-delivered flyers and emails to restaurants and bars in Texas that we located via public websites. Participants followed our flyer, posts, or emails to a 10-min Qualtrics questionnaire that asked them about a memorable experience in the service industry, their history with the occupation, their primary challenges, questions on their organizational and occupational identification, and their demographics.

Following our questionnaire sampling, Elizabeth, Emily, and Riki conducted interviews in 2021 with participants typically lasting 60–70 min, with others ranging from 90–127 min. In order to follow pandemic IRB guidelines, no interviews were held in-person, only via phone or Zoom. After informed consent, these interviews were audio recorded on the authors’ password-protected computers and saved to a university shared drive. Those recordings were then transcribed by the authors or professional transcriptionists and then deleted. Interviewees received a $10 gift card for their participation, which was funded by Elizabeth’s new faculty research funds from Texas State University. 

Our interview guide asked each participant questions based on their questionnaire and allowed us to tailor their semi-structured interviews. Interviews were the most appropriate method for our interpretive research, as it enabled us to understand their rich experiences of an understudied occupation (something we return to in our analysis).

### 3.2. Our Interview Participants

From our questionnaire, we had 234 completed responses, 162 which were genuine. Because our flyer was shared openly on social media, we identified bot responses, including repeated strange entries. Emily removed bot responses, which refers to nonsensical and A.I. responses from spam and coded non-people that came from outside the state of Texas and/or filled in odd and incorrect information. We had 152 people volunteer to be interviewed, and 80 of those were non-bot participants, meaning we determined the responses to be genuine and coming from actual people in Texas with legitimate phone numbers. Out of the 80, we selected participants through maximum variation sampling to gather multiple Texas regions, occupational positions, and intersectional experiences. We completed 38 interviews (*n* = 38). At 38 interviews, we reached data saturation, where participants provided no new exemplars [57].

While our participants largely mirrored U.S. Bureau of Labor demographics in the service industry shared above, we also faced challenges in our recruitment and sampling participants who chose to be interviewed. Many later declined to complete an interview or cancelled multiple times, leading us to have white women participants overrepresented while also having many LGBQ+ participants. We also experienced extensive “ghosting” by participants for interviews who had agreed to an interview but had to repeatedly cancel due to their chaotic schedules working and living through a global pandemic.

Moreover, we recognize how our positionalities are co-constructed throughout research [58], and white women participants’ willingness to complete questionnaires and interviews tie to our own positionalities as white, cisgender women who are U.S. citizens. We also did not have funding to recruit and interview in multiple languages. Men, trans people, nonbinary people, people of color, and/or undocumented people may have felt reluctant to be contacted and interviewed by us as white women, despite our personal experiences in the service and retail industries. Who we are and were in relationship to participants’ own evolving positionalities is not static but negotiated throughout research and in relationship to emotions (see [58]), especially in researching topics of both familial PWRs and organizational violence.

Here, we introduce our 38 interview participants’ self-described demographics (see Table 1). While we present their overall demographics as a table in order of the frequency of responses, we focused on participants’ unique, intersecting identities and experiences across our interviews and analysis. We note each participants’ salient identities as we introduce them below. We invited participants to choose pre-provided demographics in their initial questionnaire and/or self-describe their identities and also skip any question they preferred not to answer.

### 3.3. Data Analysis

After completing our interviews, we used Dedoose to analyze our deidentified interview transcripts with all participants selecting their own pseudonyms. Deidentified refers to the process we took to ensure participant confidentiality. Before interviews began, we asked participants to create their own pseudonyms and only referred to them as such. We also created unique anonymous codes for each participant to label their files to further protect their identity and removed any identifying information, including their organizations, managers, and coworkers.

We noticed during early interviews and across initial data analysis that an inductive theme of PWRs was emerging in our data, despite PWRs never being a focus of our interview guide or research questions. Communication about PWRs mostly occurred when participants responded to our interview questions about their communication with managers and coworkers and their favorite aspects and what they hoped to maintain about their occupation. Transcripts revealed repeated themes of how PWRs shaped service industry workers’ occupational experiences and their communication.

Importantly, we purposefully did not go back and add a research question about PWRs after our literature review, as we value marking the backstage of qualitative data analysis which is so often cut out of published research [59]. New themes and directions can emerge entirely from participants’ lived experiences, rather than researchers’ questions. After PWRs emerged inductively in our work, we located much of our literature review above and used a supporting call from Rumens [60] advocating for researchers to go beyond postpositivist orientations to workplace relationships theorizing to include other methodologies and theoretical orientations. He argued, following other Communication Studies scholarship, that using qualitative inquiry can “bring to the fore how the meanings attributed to these relationships are contingent on participants’ sociocultural standpoints, hierarchical positioning, and the social contexts in which they are embedded and enacted” (p. 1154). We also pair our qualitative inquiry with critical theoretical lenses to offer new occupational and methodological avenues of theorizing PWRs. Rumens’ guiding questions helped us situate our study within this growing literature, including “How do workplace friendship practices shape processes of organizing, organizational cultures, and structures?” and “How do multiple personal, social, economic, and organizational contexts influence the formation and maintenance of workplace friendship?” [60] (p. 1162).

As PWRs emerged in our data analysis, we invited Hannah to join our team, and the four of us utilized open coding on 9 initial transcripts of our 38 to surface *in vivo* and open codes. We created 100 open and *in vivo* codes across our team related to PWRs. We met extensively to compare codes, moved to secondary coding, and created a codebook for our analysis of the remaining transcripts. We finalized 14 salient “parent” codes in our codebook (such as “the love for what you do”, alcohol and drugs as PWR building, SI as family) and four “child” codes under the dark side/inappropriate PWR codes (e.g., harassment in/impacts PWRs).

After completing our coding and extensive team conversations, we continued further analysis, met to finalize our codebook, and thematized our findings. Importantly, our themes were both cultivated via coding as a team but also in dialogue together synchronously via Zoom and asynchronously via track change dialogues. Our collaborative qualitative data analysis and writing was generated through what Eger and Way [61] (p. 92) describe as, “lively connections we made together through our analysis while also illustrating how our positionality and experiences shaped how we made sense of analysis in different ways”. Our positionalities as researchers and whole people were not only co-constructed during our data collection but also in analysis, in relationship to one another and to our participants, and in writing this essay (see [58,61]). Through this analysis and writing processes, our qualitative inquiry achieved resonance, what Tracy [62] (p. 845) discusses as creating research and praxis impacts “across a variety of contexts or situations”. We also write with what Tracy [62] calls multivocality in mind as we share the rich, diverse, and connected experiences from our participants’ PWRs in service industry work, to which we now turn.

## 4. Communicating about Personal Workplace Relationships in the Service Industry 

PWRs were central to our participants’ experiences in the service industry. As we previewed above, both community and harm were created through their PWRs. We present three larger themes of: (1) fostering and sustaining PWRs, (2) the service industry as a family, and (3) PWRs as supporting inappropriate communication and organizational violence.

### 4.1. Fostering and Sustaining Personal Workplace Relationships

Across interviews, many participants discussed the importance of developing PWRs to navigate and survive in the service industry. Participants shared a communal orientation to workplace relationships that in many cases went beyond being “just coworkers” across two sub-themes. Communication with coworkers and managers worked to (1) create and foster PWRs and (2) sustain them once they were developed, including nuances unique to the relationships between employees and managers. As we introduce participants’ voices herein, we remind readers that names are their chosen pseudonyms to protect participants’ confidentiality.

#### 4.1.1. Creating and Fostering PWRs

Although almost all participants talked about creating and fostering workplace PWRs via communication, how individuals undertook these practices varied. Fostering PWRs included: extensive time spent together, open communication about personal struggles and “real life”, and elevated levels of trust due to the “dependent” nature of the work environment. Many participants first noted that just spending long hours together under difficult and chaotic working conditions and/or very slow days allowed them to create and develop friendships and romantic relationships that may not have happened otherwise. Participants repeatedly emphasized that their workplace relationships and their depth and intensity were highly influential on their experiences in the service industry. Though participants did not directly utilize the academic, formal phrase of PWRs, their discussion of these relationships align with our definition of PWRs [9]. This is exemplified by server Lilly (a white, heterosexual woman in her early 30s) when describing her communication with coworkers and managers where, “we would just sit and like, chat and talk and get to know each other on like, a deeper level than you could when you have like, a line of people”. Bartender Anton (a white, heterosexual man in his late 30s) echoed this and added that time spent together also allowed for the development of inside jokes and banter to help develop relationships. He explained, “You get a shorthand with the people you work around pretty quickly, whether it’s a sense of humor, whether it’s asking for something...Usually there’s a lot of friendliness involved. I mean you get used to being [in] very close proximity”.

Furthermore, participants expressed how open and honest communication built closer relationships that led to future PWRs. One participant, a former sous chef and manager named Cher (a white, lesbian woman in her late 30s), noted her communication approach as a restaurant manager:

*I do my best to give advice… to help people through. And that’s how I gained a lot of trust from people and a lot of friendships and stuff out of that because they felt like they could confide in me. I felt comfortable sharing if I had something relatable to share.*

Similarly, a shift lead named Sally (a half white, half Latina, heterosexual woman in her early 20s) said, “I think [being open], it’s, ultimately, a good thing, because again like that builds more meaningful relationships, if you know what people’s struggles are. And I was really grateful when people would confide in me”.

Additionally, participants described building PWRs through trust or dependence. As a bartender and server (who also worked in expo and hosting), Rachel (a white, heterosexual woman in her early 30s) described it as, “We’re very dependent on each other”. General manager Renee (a white, heterosexual woman in her early 30s) framed trust-building as essential when she stated, “you really have to have that rapport and build that trust with someone”.

Participants also created and fostered PWRs through physical touch or physical acts. This included anything from bringing food in to cheer people up to hugs and other forms of touch. The few participants who valued supportive touch spoke very highly of its importance, especially given many physical distancing mandates due to the COVID-19 pandemic and isolation. Bartender and server Rachel highlighted this with her male coworker when she stated, “In the back corner, I’ll just be like, ‘Can you just like hold me for like two seconds?’”. After a silent hug, she would “just take a breath, and it just feels like it recharges my battery, like I feel human again…And just having that two seconds of just being held…that, that means the most to me”. Thus, time spent together, open communication about personal struggles, and consensual, comforting touch allowed participants to help build relationships.

Furthermore, our analysis included PWRs across ranks with managers and employees. Some participants detailed positive, fulfilling, and healthy manager-employee PWRs. Server Callie (a white, heterosexual, gender fluid woman in her early 40s) emphasized her positive PWRs with a father-son management/owner team:

*They’re great. I mean, they take us on kayaking trips. I’ve been to their house for parties. They’ve been to my house. When I had COVID, they were calling me every day. “Can I get you anything from the grocery store?”. They were dropping off vitamins. I mean, they’re just really, really great people to work for.*

Some managers shared struggles with developing PWRs with their employees due to strict workplace guidelines, including sometimes using secrecy about friendships, while others specifically described open manager-employee friendships. Bartender Julie (a white, bisexual woman in her late 30s) shared when she found out she was pregnant during the beginning of the COVID pandemic, her management responded with the “best reception that I could have ever gotten” in the Texas Service Industry. She stated, “So I had the unique experience of I got pregnant while I was working in a bar, and [my management] said to me, ‘You know that’s fantastic, take off as much time as you want, as long as you promise to come back’”. Julie’s PWR with her manager helped support her medical and life needs. Managers and employees could thus co-create enriching PWRs.

#### 4.1.2. Sustaining PWRs

After building PWRs, participants actively worked to sustain and care for their relationships, and many PWRs made at previous service industry jobs transitioned into lifelong friendships. For participants, this primarily happened through time spent together outside of work hanging out, including outdoors, at dinners, at parties, or at bars or clubs. We noticed a common code of “alcohol and drugs as PWR building”, which could both foster play and addiction (which we explore further below). This builds upon a past national survey by Moore et al.’s [63] where young restaurant workers’ “socializing with coworkers was found to be a risk factor for heavier or problem drinking” in restaurant work (p. 331). Bartender and server Rachel described a larger annual organizational Christmas party with multiple restaurant branches that attend as “funny to go to it, because you know which group is ours, because we’re so small that we don’t leave each other’s side. Like all of our significant others that we bring to the party, we’re all in a big group”. Server Michelle (a white, heterosexual woman in her early 20s) discussed how individuals in her organization requested the same time off to coordinate and said:

*There’s a restaurant right up the road that actually serves drinks. A lot of people go meet with each other after their shift. They’ll go play at their apartment complex together, play volleyball, go tubing, go do whatever after work or make sure they request days off together to do something.*

Bartender Rosa (a white, heterosexual woman in her early 30s) also engaged in drinking with her coworkers as to maintain PWRs and destress after a long day: “At the end of the night like maybe like an hour after we closed, like I might crack open a beer every now and then, or like you know how like how somebody might have a glass of wine, like in the back or, but it isn’t like, we’re not getting shit faced, like we’re not getting drunk”. For former sous chef and manager Cher, drinking was specifically encouraged by her organization to sustain relationships. She said:

*There was a bar tab set up specifically for that reason for the managers and employees to get to hang out after work and be able to bond over that. Which, I mean, that’s where I found one of my work families; we always hung out and got a couple of drinks after work. After a long, hard shift, we would hang out together.*

Here Cher illustrated that her organization not only encouraged the development of relationships across rank, but they also offered free alcohol as a social coping mechanism after “a long, hard shift”.

Like Cher’s example, some participants spoke of drinking as a positive aspect of PWR maintenance, yet many participants also maintained PWRs through potentially risky health behaviors, such as excessive drinking, drinking at work, and/or drug use. For many interviewees, drinking was discussed as a form of coping to handle their difficult work. More importantly, drinking became a common form of socializing, especially with those in the service industry who “understood” their challenges. In turn, drinking and drug use acted as a form of relationship building for some participants in our study.

Server Callie emphasized coping and PWR building through drinking when she told us: “There’s a bar right next door, and so it’s just like a routine. You get off work, and everybody just goes to the bar and sits down and bitches about the shift”. Further, while bartender Rosa highlighted drinking as a relationship maintenance behavior above, she also discussed how drinking within service industry friendships can turn into alcoholism:

*Whenever you get off work there’s not really many places to go, except the bar… Where else are you going to go? But you want to be social, so you have to go to a bar. And there’s nothing else to do at a bar besides drink, so it just kind of happens. And the stress can be a lot sometimes… So alcoholism, you know, easy to turn to, to like destress and decompress. Again, like everyone around us doing it, so like that peer pressure. It’s really hard to not get swept up in that, I guess.*

The routines of heavy alcohol consumption became a form of PWR relational maintenance, social support, and an occupational norm for coping with organizational stress and violence.

Workers also described drug use as supporting PWRs. Lead hostess Katherine W. (a white, bisexual woman in her early 20s) recalled a previous restaurant she worked at where, “we would regularly have like Thirsty Thursdays, and I won’t name the chain restaurant, but we would like shut the restaurant down go smoke weed in the back, like hang out, do coke”. These ideas are echoed by server Landon (a Hispanic, questioning man in his early 20s) in his interview when he discussed the overall impact of drugs and alcohol as relationship building in the service industry:

*I think it just causes so much stress that all you feel like you can do in your free time is drugs or alcohol, because that’s where, that’s where all your coworkers are who are also performing this emotional labor and that’s how you decompress.*

Thus, for some of our participants, while building and sustaining personal workplace relationships was central to their job in the service industry, many highlighted tensions and potential health risks with building and managing their PWRs.

### 4.2. Service Industry as Family

Second, our inductive analysis revealed that participants commonly viewed their PWRs with coworkers and managers through what they termed a “service industry family”, which mirrored some of the communication practices detailed above under general PWRs. We name these familial personal workplace relationships (familial PWRs). Three sub-themes illustrated familial PWRs: (1) as forged in stress and labor, (2) as helping and caring, and (3) as occupational identification. We will return to a fourth sub-theme on constraints and violence created through familial PWRs in our final theme.

#### 4.2.1. Familial PWRs as Forged in Stress and Labor

First, interviewees described familial PWRs as forged via the stress and hard labor of working in their occupation. Manager Nicole (a white lesbian woman in her late 30s) shared, “I love the pace, and I love the people. I really love when it’s insanely busy, and everybody just kind of comes together, and we get through it…Everybody that I work with right now is kind of like a family. I kind of thrive off of the hectic, stressful situations, and I’m able to keep my cool”. When prompted to explain how the family relationships are cultivated, Nicole questioned if other occupations created familial PWRs due to stress, as she had only ever worked in the service industry. However, she justified the family relationships in part through the difficulties of the labor, suggesting, “I think that getting through really hard days and just helping each other out. We take turns making breakfast for each other. I spend more time with them than I do with my friends and most of my family. It’s *long* days. It’s *hard* days, and you go through it *together*”. For Nicole and other participants, it was not only the lengthy shifts together but also the difficulties of the occupation which creating spaces for care and nurturing.

Similarly, manager Marie (a white, heterosexual woman in her late 30s) echoed that familial PWRs kept her in the occupation:

*No matter how tough times have gotten, and every location that I’ve worked at—whether it’s the company I’m currently with or others—it’s always been the people that end up keeping me going. Both customers…and my employees, I always say that we’re—each of my restaurants is like my own little family pod. I really, truly take care of my people as if we are part of one family unit...Even when things were hard, I couldn’t let them down. I couldn’t leave them.*

Here Marie not only shared that her “family pod” was co-constructed in each restaurant where she worked, but also how familial PWRs are created through “tough times”. Her framing of, “I always say that we’re…a family pod”, importantly used self-reflexive communication with others about their family identification. Past research has discussed how self-reflexivity sustains shared collective identities and is also self-referential, i.e., referring to who we are as a collective [64], who we are within that collective [65] and how we cultivate and identify with family at work [13].

Later in her interview, Marie returned to how:

*going through all the hard stuff has created a need for us to be a more cohesive team, and it’s just created better relationships with us as co-managers, as well as our relationship with our team. And, it’s really, I know me and my lead manager, and I have really made it our focus at our location to create a sense of family.*

Marie credited her and other managers focusing on PWRs as central for the organization’s success. Marie’s district manager had recognized their restaurant as a space where “no matter how hard it gets we have fun…We just take care of each other. We’re like a family unit”. Here, Marie tied the difficult and hard facets of the work of creating fun despite challenges and nurturing familiar PWRs to prevent turnover, even during the pandemic. She contrasted this to other locations of her same restaurant who did not center familial values and relationships.

Finally, participants also shared the difficulties of non-service industry loved ones and the general public failing to understand their occupation and dismissing their labor as temporary, something to do while going to school, unprofessional, for youth, and optional. This bonded workers more to familial PWRs because they collectively “understood” their occupation in ways outsiders could not. Familial PWRs could also help participants challenge dirty work stigma of restaurant work [30]. Former sous chef and manager Cher said that despite two college degrees, she chose work in the back of the house as a cook because of her love for food and for the PWRs she built, including even meeting her wife at work. She shared that, “A lot of people don’t understand that it is a good job, but people work hard for it…Even my family doesn’t understand. They think it’s, I don’t know, beneath me or something”. Cher navigated the challenges of how outsiders perceived her work because she had “built up a big enough service industry family of friends, and even my own wife, that *we understand* what we do. We understand how important it is and how hard it is to make a restaurant run smoothly, and the sacrifices you have to make when you don’t get holidays; you don’t get vacations”. Coworkers in the service industry became friends and family, in part, because they understood the sacrifices of a lack of holidays or time off and the collective, exhaustive labor to run a restaurant or bar. The stresses workers encountered also connected to their familial PWRs as sustained via help and care.

#### 4.2.2. Familial PWRs as Helping and Caring

Our second sub-theme on familial PWRs were sustained through help and care and transcended purely professional relationships. Bartender and server Hank (a white, heterosexual man in his early 20s) valued “the family environment. Like a lot of times, people who work there, like servers, it does seem like a family, because you’re in the trenches together, and you’re working through it together… People will help you out, like they genuinely care about you”. Hank connected family not only through hard labor “in the trenches” (using a metaphor of war) but also through genuine care building a family.

Servers also communicated that family was cultivated through a shared emphasis on care through open communication about safety. Bartender Julie noted, “We were all like a family 100%, and so any concerns we had, [the owners] wanted to know about and were considerate of it. Especially, when the pandemic started to come about, there was open communication every single day about what was happening, and the safety of us more than anything else”. Julie connected her familial PWRs that began before the pandemic as creating open and honest communication where employees could call out sick and raise safety concerns. In contrast, she shared because Texas is an “at-will employment” state, employees could be fired without reason. Julie did not fear disclosing her pregnancy at work (as mentioned above) and asking for time off during the pandemic because of the restaurant’s familial care. In contrast, Julie warned, “There are other places where it’s like if you have a cold, and you want to call in sick, that’s just not even possible”. Thus, her experiences point to how PWRs can lead to employees’ positive or negative experiences of health and safety at work, including disclosing pregnancy or health risks and engaging in presenteeism.

Like Julie’s owners valuing relationships and care, Manager Nicole responded to our follow-up question to her mentioning family of, “How does that family type relationship gets cultivated when y’all are working together at the restaurant?” by explaining, “We ask questions about each other’s lives. We help each other out covering shifts. When stuff is going on, just doing anything we can. When people are struggling, we pull money together for them”. Unlike some organizations where there was “high turnover”, Nicole’s restaurant had many employees who had worked there from five to 20 years creating a “family”. Here, Nicole also mentioned professional tasks like covering shifts but also financial and emotional support when a coworker was “suffering”.

Server Alison (a white, pansexual woman in her early 20s) echoed the importance of working in organizations where “people kind of stick around” without large amounts of turnover and where relationships are sustained. Participants like Nicole and Alison thus equated familial PWRs with addressing turnover problems in the service industry (see [8]) and supporting past research on PWRs reducing turnover [27]. Alison also explained how familial PWRs were created through diverse identities and experiences because the work “brings a bunch of different individuals to the table from all kinds of different areas in their life, and you get…like different perspectives on everything…just like the big like family aspect of it”. Here, family was constructed not only via long term employment but inclusion for diverse and intersectional identities, which multiple participants described.

Lastly, workers created familial PWRs by learning about and caring for one another’s lives and loved ones outside of work (e.g., their romantic partners, children, and non-industry friends and families). Reservations Manager Bella (a white, heterosexual woman in her early 30s) considered PWRs as:

*Everybody’s pretty tight. Everybody’s very fluid with their conversations…We know each other’s significant others; we know each other’s children. There’s definitely an awareness of being, you know, a manager versus being an employee. But it’s more grey at the current place that I’m at rather than black and white.*

Here, Bella also shared the way coworkers and managers communication shaped relationships through using informal texting, fluid exchanges that went beyond formal roles into PWRs, and supporting each other’s loved ones.

General manager Renee similarly sustained relationships with former employees who had worked with her in their youth, stating:

*I always really treasured having someone that started so young and watch them kind of grow up…and learn a lot of life lessons in the food industry, good and bad. And kind of watch them mold themselves into young adults and go on with their lives. I still keep in touch with people that I hired 10 years ago. And to see them with their families, I’ve had team members get married, have children now…[to] have that connection with each other so much, almost more than your own family.*

Renee understood the age of her younger employees and them learning about work-life with her helped create a space where they stayed in touch and continued to care for one another’s future families. She also tied this to time spent working “almost more than your own family”, illustrating how the SI family is sustained over years. Such long-term familial PWRs led to our final sub-theme of family not being merely in one organization but across the entire occupation.

#### 4.2.3. Familial PWRs as Occupational Identification

While some participants reported how familial PWRs were created and sustained in a specific organization (and how they then identified with that organization because of those relationships), many of our participants viewed service industry PWRs as transcending one organization. Instead, service industry families were *occupational*, leading them to identify with their occupation. The familial PWRs as occupational went beyond a container of an organization and thrived over years and decades, even if organizations closed permanently. Former sous chef and manager Cher’s favorite part of her work was:

*Oh, the people! The people! You meet some really good ones; you meet some really terrible ones. But overall, you build this family feel in a lot of places that I worked. I have an amusement park family. I worked downtown at that restaurant; I have a huge work family there. Even though we no longer work together, at the airport, I still get invited to someone’s wedding [from there]. The family dynamic is very strong.*

Cher mentioned three family clusters that she maintained after she exited the organizations, even after one closed permanently. Many participants like Cher created family PWRs in each new place, even amidst toxic relationships (something we return to below).

Reservations manager Bella correspondingly reported that, “At my current job we’re all friends, it’s like a family. We’re not seeing each other outside of work, because of the pandemic and we know that if one of us gets sick all of us will then get sick. But for the most part, any restaurant I’ve ever worked at has been like that, like a family”. Here, Bella lamented not being able to have out of work time with her service industry family currently because of the COVID-19 pandemic, and she considered all restaurants part of her larger occupational family sustained for years. Bella continued:

*The camaraderie can make a shit restaurant totally worth it. You can work in a place that is serving, you know, hot dogs and Fritos, and you can still love it, because your family is there. That is such a special part of this industry, and I still keep in touch with people from all the different jobs that I worked at. These are great, smart, funny, interesting people who genuinely love each other.*

The love for one another despite informal cuisines, bad environments, high stress, and even in “total shit” as Bella said was a resounding theme for participants who viewed the occupation as sustaining familial PWRs. Server Alison shared PWRs where “you’re like super close” across multiple SI jobs, as “pretty common because we’re all kind of dealing with the same thing. Just the way we speak to each other, it definitely feels like a family. I like it a lot, honestly, the relationship with my coworkers”. PWRs, then, for many participants were common and a highlight of their occupation, building familial PWRs across space and time.

Notably, bartender and server Rachel described the “multiple forums” of service industry social media groups as sustaining familial PWRs, especially in the major cities in Texas. She explained:

*We’re a family…It’s just having this brotherhood, and this family if somebody is having a problem, or they have questions about Texas Alcoholic Beverage Commission or something like that. We have people that have been in the service industry for 40 years or something. And just keeping that family no matter what.*

Rachel shared that despite “high school” dynamics and drama among PWRs and current COVID-19 pandemic trauma, “we’re still family”. Social media, then, became a place where family could be constructed across the occupation.

Finally, we found an *in vivo* theme “the love for what you do” as a form of occupational identification connected to familial PWRs. This initial code came from a quote from former sous chef and manager Cher:

*I’d say the love for what you do, because I know the back of the house, some people cook just because it’s a job; some people cook because they’re passionate about it. Front of the house: some people love actually connecting with people, and making money, and having that conversation. A lot of people are great at conversations with people, and a lot of bartenders, a lot of servers, and that and the whole family feel like when you get a good crew together that works together and helps each other, that’s the best feeling.*

Cher listed how different restaurant divisions of labor created unique expertise and connected everyone together for a “whole family feel” who loved and identified with the occupation.

Many participants echoed Cher’s “love for what you do” code. Others reported occupational identification because of their service industry occupational familial PWRs as: “loving food” as a joy together (Nicole, Marie), a “labor of love” working together (Bella), loving the “chaos” (Nicole, Cher), “sweaty hard work” accomplished with your family (Callie), and a love and passion created via customer and coworker PWRs (Kayla, Zee, Jim). The unique familial PWRs cultivated in the service industry across the occupation was sometimes cited as the only redeeming feature of a toxic, hard industry. We now turn to how PWRs not only created love, support, and family in the service industry but also were mobilized to perpetuate organizational violence.

### 4.3. PWRs as Supporting Inappropriate Communication and Organizational Violence

Our final section examines inappropriate and dark side communication themes from our analysis. First, we explore the potential for sexual harassment, bullying, and harassment present in PWRs. Second, we introduce problematic manager-employee PWRs subthemes.

#### 4.3.1. Service Industry PWRs Cultivating Sexual Harassment, Bullying, and Harassment

Although many participants fostered long-lasting relationships, our analysis surfaced a dark side of PWRs. The following participants shared experiences where originally positive, consensual PWRs with coworkers and managers shifted to inappropriate relationships and/or violence. Server and trainer Mary (a white, heterosexual woman in her 20s) reported a time when she newly became friends with a coworker who later asked her to start a more intimate relationship. When she declined, “He started to ignore me, treat me like shit, snide remarks, like all this stuff”. She no longer felt comfortable around this coworker that she had previously bonded with. Then, when she tried to report his bullying, her manager’s response was to fire him, which Mary declined. Instead, she dealt with the bullying until one day she “just fucking screamed at him in the middle of the floor, [and] was like, ‘Shut the fuck up, like, leave me alone! You won’t just leave me alone’”? Mary’s once-positive PWR turned into daily bullying, leading to her resistant screaming.

Another participant, bartender and server Gerald (a white, heterosexual man in his early 20s), had friendships with multiple coworkers of different genders, and they would all go out for drinks together after work. He reported that over time he would notice some coworker-friends “giggling on the other side of the room”. When Gerald asked what they were laughing at, they said they thought he was cute; later he found out that they were actually “trying to see if they could get [him] to cheat on [his] girlfriend”. He felt betrayed and immediately ended his friendship with those coworkers. Gerald did not report this to his managers and said he had to “be professional” with them for another year after the incident.

Many other participants experienced betrayal in PWRs with coworkers or managers they assumed they could trust over time, only to be bullied or sexually harassed in the future. We heard examples including: to-go server and bartender Zee (a white, heterosexual man in his late 20s), whose coworker started to “grab [his] penis” out of nowhere; front of house manager Tekla (a white, pansexual woman in her early 30s), whose coworkers would comment on “like how tight [her] shirt was”; or bartender Betty (a white, heterosexual woman in her early 30s), whose manager asked, “are you blowing him?” when she was closing with a close coworker one night. These participants all thought they could trust their PWRs, with coworkers and managers alike, only to later experience harassment through those relationships.

#### 4.3.2. Management and Employee PWRs as Organizational Violence 

In coding, we specifically created a sub-theme for management and employee PWRs, and within that code, we surfaced examples where managers harnessed organizational violence against their employees, including victim-blaming, abuse of power, negligence, harassment, sexual harassment, discrimination, bullying, retaliation, and more. We included sub-themes of: (1) communicating emotions within manager PWRs, (2) manager-employee PWRs and dating, (3) manager PWRs supporting organizational violence, and (4) organizational violence through familial PWRs.

**Communicating Emotions Within Manager PWRs.** First, participants described their required emotional labor to manage organizational violence within employee and manger PWRs. Server Symone (a Black, heterosexual woman in her mid-20s) shared her experience in performing emotional labor with management. She said, “There would be times when a manager would like legit come, storming [into] like the kitchen area, just like flying off the handle … and I’ll just be like ‘What is wrong with you?’ Like, I would go up to them and say like, ‘Hey, I understand that you’re frustrated, but you cannot like sit up here, this is not how you run the shift’”. Here, Symone identifies her frustration with her manager’s inability to control their outbursts, taking on a role of emotionally managing her manager’s violent communication.

Similarly, server Landon also experienced, “If managers were in a bad mood and you didn’t perform or answer their questions in a certain way, and they snapped on you, like that’s just what would happen. Like, I feel like my conversation was dependent on how happy my manager was that day … [Management] can take out their emotion on you and that directly affects your job”. Landon referenced the power that service industry managers can hold over their employees. Landon and other participants described how managers decide which shifts are scheduled, which can dramatically impact the amount of money workers take home in tips. Additionally, managers assign tables to servers that are “good” (seat more people, meaning a large check and hopefully a large tip), or less desirable tables (tables that only seat two guests, fewer tables). Because of the emotional volatility of Landon’s manager, he was careful to manage his own emotions to prevent impacts on his income.

Often, manager-employee PWRs resulted in emotional manipulation. Server and manager Katherine P. (a white, bisexual woman in her late 20s) detailed an experience in which her service industry friend quit a job because the friend’s manager (who also had a PWR with Katherine’s friend) was mistreating her. The result of Katherine’s friend putting in her two-week notice was a sobbing voicemail from her manager. Katherine shared with us her advice to her friend:

*I was like honestly, you should tell her [the manager] that we can only talk through HR because we have a dual relationship, and I’m now worried about damaging our friendship and my future in this industry, because you’re my boss, and you can stamp out my career as a pastry chef, you know? This grown woman [manager] is all upset that she treated someone so poorly that they’re quitting and now… this grown woman is asking the person that’s fucking quitting to like take care of her… This is still a professional relationship.*

Here Katherine identifies the inability of her friend’s manager to separate their working relationship and their friendship. This inability is so pronounced, her friend’s manager left her a lengthy, unprofessional voicemail.

Additionally, to-go server Karlie (a multiracial, heterosexual woman in her early 20s) also was damaged by her perceived PWR with her manager as it changed when she got an additional serving job. Karlie’s manager appeared to almost be jealous of Karlie’s other position and would be passive aggressive towards her while at work. One day, Karlie was overwhelmed with her manager’s minimizing communication and broke down in tears. Karlie’s other manager approached her to see if she was alright and Karlie stated, “Like, I had to like make up some story about like my mom or something. I don’t even remember what I said, but she was just like you know, like ‘The other manager like she’s just, she’s just hard on you, because she loves you’ and blah blah blah. So I just had to be like ‘uh huh yeah, uh huh’”.

Here we see Karlie’s other manager using “love” as a justification for emotional manipulation, a tactic that is all too common in the service industry (see [30,66]). Karlie’s experience with emotional manipulation aligns with past research theorizing workplace friendships and relationships [10,67]. Workplace friendships and relationships, which are often created because workers have a dearth of spare time to build connections outside of work, can result in control over workers [10,67]. Coworkers and managers justifying emotional manipulation and requiring emotional labor also aligns with authors Emily and Riki’s innumerable personal experiences with emotional manipulation while working in the Texas service industry.

**Manager-Employee PWRs and Dating.** Second, some workers also described situations where managers would be in romantic relationships with their staff, which led to favoritism and abuse in the organization. Server Callie shared, “And there was one girl I worked with in particular, and she was dating the owner, and she was awful. She was a bully”. Callie then continued to describe a situation where the woman she worked with who was dating the manager would bully Callie into trading shifts with her so that the girlfriend of the manager would have better shifts that typically came with more money. Callie lamented, “It’s like she’s dating the owner. You can’t complain to him about it”. Callie was frustrated because the person she would go to, typically, to report situations of bullying was one of the people complicit in her experience with bullying.

To-go server Karlie also told us about when one of her service industry friends was dating a district manager, only to find out that the district manager was utilizing his work travel to have multiple sexual relationships with other women employees. When asked if she or her friend reported this behavior, Karlie said, “since he was a higher up, he was like sort of friends with everyone on HR and stuff like that, so she just didn’t feel like it would get anywhere”. These excerpts involved (assumed) consensual romantic relationships between management and employees, though power was at play. The following section delves deeper into the dark side of manager-employee PWRs, which can present instances of harassment and bullying.

**Manager PWRs Supporting Organizational Violence**. Third, participants narrated violence they experienced through their PWRs with managers. Server Mariah (a white, bisexual woman in her early 20s) detailed a graphic incident of sexual harassment from an owner that also would drink while on the clock. This owner sexually harassed all the women working during his shift, and her female coworkers were hesitant to report the sexual harassment:

*All of them were like “No, it’s not a big deal”, and they’d like come up with a nickname for him and be like, “It’s just blank”, you know. And I’m like, you’re enabling him first of all. And also you’re just letting this horrible environment be horrible and not just for you, but for everyone else here. And if you call him by this nickname, like fondly after something like that, then that’s just going to encourage him, you know? And maybe they’re just trying to remember the “good person” that they know, on a personal level so that they don’t feel like it’s just someone being creepy. But, I don’t have that sentiment.*

Mariah communicated frustration with her coworkers’ refusal to confront their harasser, choosing instead to give him an endearing nickname presumably to recenter their PWR with their harasser.

Bartender and server Rose (a Hispanic, asexual woman in her late 30s) shared an instance where after repeated sexual harassment from a coworker, she reported the sexual harassment to a manager that she perceived she had a PWR with. However, when Rose ultimately decided to report, one of her managers made her feel less than, “So I ended up telling a manager. She’s like an acquaintance... so I felt comfortable in telling her, otherwise I don’t even know if I would ever spoke up”.

After Rose reported her experience with sexual harassment to her “acquaintance” manager, and her harasser was fired, Rose experienced additional victimization from another one of her female managers. This other female manager made Rose feel, “like maybe I had did something to bring [the sexual harassment] on… I just remember feeling … I don’t know, I guess, like a victim as much as I don’t want to say it. I just, I felt so victimized by her. Like she didn’t believe me”. Here Rose identifies her disappointment in the route her management team chose to handle her experience with sexual harassment from a coworker, especially in her other woman manager who she had assumed gender solidarity with. One of the primary reasons Rose chose to report her experience was due to a PWR with one of her managers, though that feeling of closeness was disrupted by her manager’s reaction.

Unfortunately, this shattering of a perception of PWRs with management was common for our participants. Typically, interviewees described initially feeling close and building a PWR with a manager. Then when a kind of crisis or experiences of organizational violence arose, the perception of the PWR would be disrupted when managers would either refuse or fail to appropriately respond to the crisis. Managers thus mobilized PWRs in using inappropriate communication and thereby structured organizational violence, including in familial PWRs.

**Familial PWRs as Organizational Violence.** Fourth and finally, participants shared how managers used what we identified as unique familial PWRs as “performative” and to perpetuate and harness organizational violence. Food truck manager Lilly recounted the harassment she and her coworkers faced from an upper-level manager who drank on the job at work, “would really degrade the employees”, and how it mirrored harassment happening “so often” across the occupation that it was solidified as “an unfortunate part of the industry”. Lilly formed PWRs with her coworkers in part because of the abuse they faced together. She explained that her employees “would tell me—and we became very close—and it kind of became, like this is so sad, but it kind of became a joke among the employees that he was like our drunk alcoholic dad that we just wanted to make him happy, [and] we couldn’t”. She shared that the “mentality of the team” was to “try to avoid him, or just be really careful when he’s around and then, when he wasn’t around the job, which is like exponentially better”. When asked why employees began comparing him to a drunk, alcoholic dad, Lilly explained:

*Whenever my coworkers said it one day, and I was like, “Oh my God, you hit the nail on the head”. That’s exactly what it’s like it’s like. You cannot please him, nothing that you do will please him so, it’s just kinda like constantly trying to do something that you know is never going to happen and then at that point just sort of trying to avoid it, and do your job, and just go home.*

Here, their manager’s harassment created PWRs among employees and lower-level managers where they used a metaphor to understand his harassment as an alcoholic parent.

Like Lilly, other participants lamented how familial PWRs supported harassment and discrimination. We share an extended example from server Landon’s past when he was 16, and many of his older female colleagues referring to him as “their work husband, boo, and cutie”. Landon explained that his managers and coworkers would share personal information with him about their negative romantic relationships. He said, “I thought it was just endearing at first…until later I was like, ‘Oh I’m a child, and these are adults who have lived through multiple relationships, where I had never had a serious relationship.’ You know I come into work, and I get my butt pinched or slapped”.

In addition to the overall experience of multiple older women coworkers sexually harassing him, Landon also shared how one of his managers in her late 20s befriended him and other youth, where they would hang out after work, partaking in drinking alcohol and smoking marijuana with the manager. One night after work:

*She [the manager] started crying out of nowhere ... telling my friends and coworkers at the time that she was sad that she couldn’t have sex with me. And at that time I was a virgin, and she was late 20s or early 30s. Which, you know, doesn’t matter too much, but she was significantly older than I was .... I don’t think I carried a lot of trauma from that, but it did make me feel very uncomfortable in the situation. And like all of a sudden, I was like, why am I drinking alcohol as a minor in front of my manager, who is physically upset because she expressed her wishes to have sex with me?*

Because Landon had previously considered his manager to be his friend, this moment caused him to question what had been normalized about manager-employee PWRs with minors at work.

Landon went on to specifically connect familial PWRs as supporting and upholding sexual harassment. When asked what “magic wand” solution he would seek for the industry, Landon responded for managers to, “really take care of their employees. And not take care of it in that way that they call it a family, because I feel like they call their restaurant a family, just so they can take advantage of them, and you feel like you can’t do anything about it”. Throughout his interview, Landon returned to the idea of working 10+ h shifts creating comfort with one another that creates, “openness to any advancement, whether they feel like they’re joking or not, because you do kind of feel like this weird dysfunctional family in a way. But a dysfunctional family that like touches each other, so like it’s very weird”. Here, Landon showed familial PWRs as supporting organizational violence and hostile work environments. Finally, Landon argued, “They say, ‘It feels we are a family.’ But I feel like family is kind of a scapegoat of saying, ‘Hey, we can mistreat you sometimes, and you’re going to forgive us for that’”. Therefore, Landon and other interviewees showcased how familial PWRs can be mobilized for mistreatment, discrimination, and sexual harassment.

Like Landon, server Symone shared how family is also utilized in restaurants to create occupational segregation that places women into public-facing roles for gendered and sexualized performances. She quipped sarcastically, “I think the whole like. ‘Here we’re a family.’ I’m like okay, ‘No. We’re *not*.’ Knowing that, I think it just promotes better working cultures”. For Lilly, Landon, Symone, and others, rather than resting on familial PWRs to hide inequities or support abuse, the service industry needed to address the harm mobilized through PWRs and how PWRs may actually maintain organizational violence. While some manager and employee PWRs can lead to long-lasting, positive, fulfilling, and even familial relationships, our participants also illustrated how these relationships can require emotional labor and sustain bullying, harassment, and sexual harassment.

## 5. Contributions and Future Directions

Our qualitative inquiry on the Texas service industry surfaced inductive themes that provide new contributions to ongoing interdisciplinary research on PWRs. Our service industry worker and manager participants credited PWRs as central organizational and occupational relationships shaping their identification with one another and their occupation, even developing what we name here as familial personal workplace relationships (familial PWRs). Simultaneously, these same familial PWRs could also become inappropriate and dangerous, where relationships could be used to harness, embolden, and cover organizational violence. Our essay yields theoretical contributions, limitations, and calls for future research.

### 5.1. Theoretical Contributions and Future Directions

First, much work-life and organizational communication research focuses on corporate or white-collar organizations (e.g., [1,2,3]) and rarely situates scholarship in blue-collar or service work (see [30,36], for recent exceptions). Researchers may also avoid studying a specific occupation, often to increase generalizability of the findings. While prior work-life and PWR literature helps theorize interconnections between our organizational and personal lives, not all organizations and PWRs function similarly. Thus, we need research to explore nuances that may exist within certain occupations that are foundational for understanding differences in PWRs. Our essay offers one example of such scholarship.

Unlike white-collar jobs where individuals may work in separate work stations or even from home, those in the service industry work closely with many other individuals throughout their shift and may work with different people every single day depending on how workers are scheduled. While our findings highlight similarities to previous research in regards to some of the hierarchical nature of PWRs (see [9,68]), in general, the service industry allows for individuals to interact and form PWRs in a way that is underrepresented in existing research. We extend past research on how customer service work and restaurant work occupational norms are communicated collectively including long hours, occupational stress, demanded emotional labor, shared challenges, and combatting dirty work stigma [8,30,53]. We specifically showcase how PWRs among service industry workers are cultivated in part through sustaining occupational norms. For some participants, PWRs were central to their lives outside of work and lasted even when they changed jobs. For our participants in the service industry, some elements of relationship escalation found by Sias and Cahill [17] such as proximity, shared tasks, and life events appeared in their relationship building. Given their high stress environment, which has been exacerbated during the global COVID-19 pandemic, we found unique communication interactions that connected and sustained service industry PWRs. Future studies may also consider distinctions of occupational segregation shaping PWRs and how relationships are formed in what service industry workers call the “front of the house” (e.g., hosts, bars, and dining rooms) and the “back of the house” (kitchen, cooks, food storage, and cleaning spaces).

Second, our research marks the complexities of PWRs as both caring and harmful relationships. Throughout our findings, we saw paralleled supportive communication in PWRs alongside inappropriate, even violent experiences. While we have robust knowledge on how PWRs are formed (see [9]), we know far less about what Long [39] calls inappropriate components of these relationships and need continued research on areas like relationship termination [21,26], information manipulation with coworkers in workplace romances [68], what happens when relationships at work end [40], and workplace bullying [43]. While our findings highlight positive aspects of PWRs, many communication practices in PWRs that we found are forms of dark side communication. As argued by Spitzberg and Cupach [38], the “dark side” and the “light side” of relationships can become so entangled that it can be difficult to separate the two. Further, behaviors that were once considered positive can very quickly become aversive [38]. Researchers should also consider Long’s important [39] critiques of dark side and light side metaphors as racialized and work to create new language for describing how relationships become inappropriate and even dangerous.

Our contribution to dark side and inappropriate communication frames is to connect to organizational violence [44] to theorize how both relationships and organizational structures can sustain harassment, bullying, and other forms of violence at work. In particular, our research adds to ongoing scholarship on how violence is normalized in certain occupations [49,50,51], showcasing a need for continued research at an occupational level. We suggest future scholarship to theorize occupational violence, which we conceptualize as how members of specific occupations create, maintain, or challenge communication practices and norms that structure the normativity of violence at work.

Our findings also directly enrich the theorization of PWRs by highlighting their complexities and complicated trajectories, such as how fostering relationships via spending time together outside of work can move toward drinking as a primary form of coping to alcoholism and drug addiction. Future research could not only identify the work and health risks of the service industry and other occupations with drinking (such as [63]) and coping with occupational stress [8] but also identify how such patterns become normative to offer potential health interventions. Similarly, PWRs could create experiences of friendship, caring, intimacy, and romance or in contrast, support organizational violence and sexual harassment. For some participants, then, PWRs that were central to their ability to work and cope in the service industry were also relationships that led to potential addiction or violence.

Third, our research extends past scholarship on organizations as families [15,16] and organizational identities and identification as sustained through construction of “chosen families” [13]. Participants communicated what we term familial PWRs in the service industry, that is forming familial personal workplace relationships that they create and sustain in their organizations and across their occupation. Most literature on work as family (as metaphor, as identification, or as an identity) connects the family to a specific organization (i.e., members of the organization view themselves as a family). This literature has not specifically addressed PWRs, which our study uniquely adds. Furthermore, while our study did reveal familial PWRs creating organizational identification, we also showed that participants uniquely saw familial PWRs as sustaining occupational identification. Many identified with being a part of the occupation and remained in the service industry because of their familial PWRs across the occupation. Future scholarship should explore familial PWRs at an occupational level and consider similarities and distinctions in other occupations tied to specific communication tensions and norms. 

Moreover, when our participants self-categorized their relationships as familial, such familial PWRs also not only sustained close relationships of care but also were utilized for organizational violence. Coworkers and managers used PWRs as justification for emotional labor, manipulation, and abuse. Such complexities are further highlighted when looking at familial PWRs as organizational and/or occupational violence. Our participants discussed how they formed relationships as employees who were being harassed by the same coworker(s) or manager(s). In a study on organizational injustice and coworker relationships, Sias and Jablin [69] found that when coworkers see others experiencing differential treatment, those not receiving special treatment turn towards each other and bond over the unfair treatment. Our results support this finding and highlight the importance of having familial PWRs with others navigating difficult, uncomfortable, or potentially dangerous situations. Overall, the complexities of familial PWRs, organizations as families, and family metaphors of organizations and occupations as both supportive and violent communication (sometimes simultaneously) needs further inquiry.

Finally, we noticed how some of our white and/or heterosexual participants had more positive experiences with PWRs (especially with managers), while many people of color and/or LGBQ+ people had more negative and inappropriate experiences with manager-employee PWRs. These experiences hinge on the characteristics of organizational violence, which further disciplines and impacts non-normative bodies (see [46,48]). Participants navigated PWRs in relationship to their experiences of organizational violence, which connected to other structures of violence like racism, sexism, ableism, and homophobia. Additional research should be conducted with service industry workers’ experiences of organizational violence from their intersectional identities. We also recommend future research on service work be conducted in multiple languages to capture the often-invisible immigrant and undocumented labor, especially in restaurant, bar, and hospitality work. Because “communication organizes difference” [70] (p. 2) and intersectionality shapes our occupational experiences, we need further scholarship on how interconnections of race, sexuality, class, age, disability, nation, gender, and more identities and experiences impact PWRs as both supportive and violent.

### 5.2. Limitations and Conclusions

Finally, while our interviews created space for participants to share their voices and lived experiences in the Texas service industry, there are a few limitations to our essay. First, some readers may view that PWRs were not the initial focus of our study as a “limitation”; as interpretive researchers, we challenge this impulse and actually recognize this as a strength where our participants illuminated themes we did not anticipate. Our interview guide emphasized occupational challenges like bullying, emotional labor, harassment, and the COVID-19 pandemic. It was through examining the lived experiences of our participants that our team connected their interviews to PWRs. We express our gratitude to our participants for their knowledge to extend theories and improve future occupational practices. 

Second, we faced limitations in our data collection. We experienced incredible recruitment difficulties to have participants complete the questionnaire and subsequently make time for interviews due to the ongoing global COVID-19 pandemic. Participants during our recruitment were overworked, burned out, and understandably did not wish to spend down time discussing traumatic subjects. Third, because of the pandemic, we were unable to travel and engage with in-person recruiting or interviewing, which we think would have helped us capture even more regions of the state and further complexities on PWRs.

Fourth, our team worked to oversample for people of color, queer people, immigrants, and people with disabilities to bring experiences often devalued in research to the forefront. While we were able to recruit many LGBQ+ participants and were able to mostly mirror many racial demographics of the overall service industry [56], we hoped to include more intersectional experiences in our study. Future service industry research should seek funding and/or be driven by team members who are people of color, trans and nonbinary, undocumented workers, and multilingual to help recruit participants, including those who are not English speakers and to invite participants to be interviewed in their preferred language. 

Fifth, as described in our methods, we chose to focus on the Texas service industry because of the near constant shifting of COVID-19 protocols and state specific mandates in the USA. We also wanted to center experiences of communities and occupations we are part of and/or have supported. Future studies should examine the service industry in other states, regions, and countries. The service industry is the backbone of our country and many more globally. Additionally, we encourage future studies of the working conditions and wages as impacting workers’ organizational and occupational experiences and PWRs. As the hourly wage for service industry workers in Texas is $2.13 [6] and wage compensation was not a central focus of this study, future scholarship should examine how work environments and compensation reflect the valuing of work(ers) and the impact of working for tips on occupational experiences and organizational communication. 

In closing, our research amplifies the experiences of care, comfort, family, harm, and violence entangled in personal workplace relationships in the service industry. We hope other scholars will join us in theorizing such complexities of PWRs in future occupations, organizations, and countries.

## Figures and Tables

**Table 1:** Participant Demographics.

		N	Percent of Total
**Age**			
	18–24	16	42.1%
	25 to 34	13	34.2%
	35 to 44	8	21.1%
	45 to 49	1	2.6%
**Gender**			
	Woman	29	76.3%
	Man	7	18.4%
	Agender	1	2.6%
	Gender Fluid	1	2.6%
**Sexual Orientation**			
	Heterosexual	23	60.5%
	Bisexual	4	10.5%
	Lesbian	3	7.9%
	Pansexual	3	7.9%
	Asexual	1	2.6%
	Questioning/Unsure	1	2.6%
	Queer	1	2.6%
	Asexual and Heterosexual	1	2.6%
	Questioning and Straight	1	2.6%
**Race and/or Ethnicity**			
	White	28	73.7%
	Black and/or African	3	7.9%
	Hispanic and/or Latina/o/x	3	7.9%
	Half White/Half Latina	1	2.6%
	Korean and White	1	2.6%
	White and Hispanic	1	2.6%
	American of European Descent	1	2.6%
**Social Class**			
	Middle Class	14	36.8%
	Working Class	11	28.9%
	Lower Middle Class	7	18.4%
	Upper Middle Class	4	10.5%
	Lower Class	2	5.3%
**Annual Income**			
	Less than $49,999	19	50%
	$50,000 to $99,999	12	31.6%
	Greater than $100,000	7	18.4%
**Education Level**			
	Bachelor’s Degree	20	52.6%
	Some College but no Degree	12	31.6%
	High School Degree or Equivalent	2	5.3%
	Less than a High School Diploma	1	2.6%
	Associate Degree	1	2.6%
	Working on a Master’s Degree	1	2.6%
	Master’s Degree	1	2.6%
**Employment Status**			
	Currently Employed in the Service Industry	30	78.9%
	No Longer Employed in the Service Industry	8	21.1%
**Length Working at Current Location for Those Currently Employed**			
	13 Months to 3 Years	12	40%
	0 Months to 1 Year	10	33.3%
	37 Months to 5 Years	5	16.7%
	More than 5 Years	3	10%
**Length Since Last Working in Service Industry for Those No Longer Employed in Service Industry**			
	Less than 1 Year	6	75%
	13 Months to 2 Years	2	25%
**Length Working in Service Industry Total**			
	More than 4 Years	26	68.4%
	25 Months to 4 Years	7	18.4%
	6 Months to 2 Years	5	13.2%
**Number of Locations Worked in Service Industry**			
	1 to 4 Locations	23	60.5%
	5 to 8 Locations	9	23.7%
	9 or more Locations	6	15.8%
**Job Type in Service Industry**			
	Various Managerial Roles	14	36.8%
	Bartender	9	23.7%
	Server	9	23.7%
	Barista	1	2.6%
	Cashier	1	2.6%
	Host/Hostess	1	2.6%
	Sous Chef	1	2.6%
	Waitress	1	2.6%
	Did Not Identify	1	2.6%

## Data Availability

To protect participants’ confidentiality, our data are not publicly available. Inquiries about our data can be sent to the corresponding author.
